# Association between age at diagnosis and all-cause mortality in type 2 diabetes: the Renal Insufficiency and Cardiovascular Events (RIACE) Italian Multicenter Study

**DOI:** 10.1007/s00592-024-02294-1

**Published:** 2024-05-07

**Authors:** Martina Vitale, Emanuela Orsi, Anna Solini, Monia Garofolo, Valeria Grancini, Enzo Bonora, Cecilia Fondelli, Roberto Trevisan, Monica Vedovato, Giuseppe Penno, Antonio Nicolucci, Giuseppe Pugliese, for the Renal Insufficiency And Cardiovascular Events (RIACE) Study Group*

**Affiliations:** 1 Department of Clinical and Molecular Medicine, “La Sapienza” University, Via Di Grottarossa, 1035-1039, 00189 Rome, Italy; 2 Diabetes Unit, Fondazione IRCCS “Cà Granda - Ospedale Maggiore Policlinico”, Milan, Italy; 3 Department of Surgical, Medical, Molecular and Critical Area Pathology, University of Pisa, Pisa, Italy; 4 Department of Clinical and Experimental Medicine, University of Pisa, Pisa, Italy; 5 Division of Endocrinology, Diabetes and Metabolism, University and Hospital Trust of Verona, Verona, Italy; 6 Diabetes Unit, University of Siena, Siena, Italy; 7 Endocrinology and Diabetes Unit, Azienda Ospedaliera Papa Giovanni XXIII, Bergamo, Italy; 8 Department of Clinical and Experimental Medicine, University of Padua, Padua, Italy; 9 Center for Outcomes Research and Clinical Epidemiology (CORESEARCH), Pescara, Italy

**Keywords:** Type 2 diabetes, All-cause mortality, Age at diagnosis, Diabetes duration, Complications

## Abstract

**Aims:**

It is unclear whether type 2 diabetes diagnosed in young adulthood is associated with increased severity than that occurring later in life beyond longer lifetime exposure to hyperglycemia. This study aimed at assessing the independent association of age at type 2 diabetes diagnosis with all-cause mortality.

**Methods:**

This prospective cohort study enrolled 15,773 Caucasian patients with type 2 diabetes in 19 Italian centers in 2006–2008. Cardiometabolic risk profile and presence of complications and comorbidities were assessed at baseline and participants were stratified by quartiles of age at diabetes diagnosis. All-cause mortality was verified on 31 October 2015.

**Results:**

Valid information on vital status was retrieved for 15,656 participants (99.3%). Patients in the lowest quartile had the longest diabetes duration, the worst glycemic control and the highest prevalence of insulin treatment, obesity, atherogenic dyslipidemia, and smoking habits. All complications were inversely associated with age at diabetes diagnosis after adjustment for age and sex, but not after further adjustment for diabetes duration. Percentages of death, Kaplan–Meier estimates, and unadjusted hazard ratios and mortality rates increased from the lowest to the highest quartile. In contrast, when adjusting for age and sex, participants falling in the lowest quartile, showed the highest mortality risk [hazard ratio 1.321 (95% confidence interval 1.196–1.460), *P* < 0.0001]. However, differences among quartiles disappeared after adjustment for diabetes duration, complications/comorbidities, or other cardiovascular risk factors.

**Conclusions:**

Type 2 diabetes onset in young adulthood is associated with increased mortality that is mainly driven by longer diabetes duration favoring the development of complications.

*Trial registration*: ClinicalTrials.gov, NCT00715481, retrospectively registered 15 July, 2008.

**Supplementary Information:**

The online version contains supplementary material available at 10.1007/s00592-024-02294-1.

## Introduction

According to estimates from the Global Burden of Diseases, Injuries, and Risk Factors Study, the age-standardized prevalence of diabetes, mostly type 2 diabetes, has increased worldwide by 90.5% between 1990 and 2021 and is expected to further increase by 59.7% between 2021 and 2050 [1]. However, the rising trend in diabetes prevalence is less pronounced in high-income countries such as Europe, where the incidence of diagnosed diabetes is stabilizing or declining [2]. In this scenario, there has been also an increasing trend in the prevalence of type 2 diabetes in adolescents and young adults alongside the obesity epidemic, though again only a modest increment was seen in Europe [3, 4].

Early-onset type 2 diabetes, generally defined as diagnosed before age 40-to-45 years, is a matter of concern because it has been shown to be associated with increased mortality from any-cause, cardiovascular disease (CVD) and non-CVD as well as with increased incidence of CVD events and microvascular complications [5–12]. However, it is unclear whether people with early-onset type 2 diabetes develop complications more rapidly than those with late-onset disease, i.e., irrespective of the longer lifetime exposure to hyperglycemia and other CVD risk factors [3, 13]. Indeed, this seems the case in children and adolescents diagnosed with type 2 diabetes, as risk was shown to increase steadily over time, with complications affecting most of these people by the time of young adulthood [14], and to be higher than in coeval individuals with type 1 diabetes [15–18]. In contrast, there is no conclusive evidence for type 2 diabetes occurring in young adults, also because this age group is under-represented in prospective and randomized clinical trials [19].

The present analysis aimed at assessing the independent association of age at type 2 diabetes diagnosis with all-cause mortality in the large cohort of the Renal Insufficiency and Cardiovascular Events (RIACE) Italian Multicenter Study, which included individuals diagnosed in young adulthood.

## Methods

### Study design and participants

The RIACE Study was an observational, prospective, cohort study on the impact of estimated glomerular filtration rate (eGFR) on morbidity and mortality in individuals with type 2 diabetes [20].

It enrolled 15,933 Caucasian adult patients with type 2 diabetes (after excluding 160 individuals with missing or implausible values), consecutively attending 19 hospital-based, tertiary referral Diabetes Clinics of the National Health Service throughout Italy in the years 2006–2008. Exclusion criteria were dialysis or renal transplantation.

### Baseline data

Baseline data were collected using a standardized protocol across participating centers [20].

Participants underwent a structured interview to collect the following information: current age, family history of diabetes, smoking status, physical activity (PA) level, known diabetes duration, severe co-morbidities, and current treatments including glucose-, lipid-, and blood pressure (BP)-lowering therapies.

Body mass index (BMI) was calculated from weight and height, whereas waist circumference was estimated from log-transformed BMI values. Then, BP was measured with a sphygmomanometer with the patients seated with the arm at the heart level.

Hemoglobin A_1c_ (HbA_1c_) was measured by HPLC using DCCT-aligned methods, whereas triglycerides and total and HDL cholesterol were determined in fasting blood samples by standard colorimetric enzymatic methods. Then, non-HDL cholesterol level was computed by subtracting HDL cholesterol from total cholesterol, whereas LDL cholesterol concentration was estimated using the Friedewald formula, i.e., LDL cholesterol = total cholesterol − HDL cholesterol − (triglycerides/5) (in mg/dl).

The presence of diabetic kidney disease (DKD) was assessed by measuring albuminuria and serum creatinine, as previously detailed [20, 21]. Briefly, albumin excretion rate was obtained from 24-h urine collections or calculated from albumin-to-creatinine ratio in early-morning, first-voided urine samples; albumin concentration in urines was measured by immunonephelometry or immunoturbidimetry, in the absence of interfering clinical conditions. Serum (and urine) creatinine was measured by the modified Jaffe method, traceable to IDMS, and eGFR was calculated by the 2009 Chronic Kidney Disease Epidemiology Collaboration equation. Patients were then assigned to one of the following DKD phenotypes [22]: no DKD, albuminuric DKD with preserved eGFR, non-albuminuric DKD, or albuminuric DKD with reduced eGFR.

The presence of diabetic retinopathy (DR) was assessed in each center by an expert ophthalmologist by dilated fundoscopy [23]. Patients with mild or moderate non-proliferative DR were classified as having non-advanced DR, whereas those with severe non-proliferative DR, proliferative DR, or diabetic macular edema were grouped into the advanced, sight threatening DR category. DR grade was assigned based on the worse eye.

Previous major adverse CVD events, including myocardial infarction, stroke, foot ulcer, gangrene and non-traumatic amputation, and cerebrovascular, carotid, and lower limb revascularization, were adjudicated based on hospital discharge records by an ad hoc committee in each center [24].

### All-cause mortality

The vital status of study participants on 31 October 2015 was verified by interrogating the Italian Health Card database (http://sistemats1.sanita.finanze.it/wps/portal/), which provides updated and reliable information on all current Italian residents [25].

### Statistical analysis

Data are expressed as mean ± SD for continuous variables and number of cases and percentage for categorical variables. Patients were stratified by quartiles of age at type 2 diabetes diagnosis (from earliest-onset to latest-onset). Comparisons among quartiles were performed by one-way ANOVA or Kruskal–Wallis test, according to the parametric or non-parametric distribution of continuous variables, and Pearson’s χ^2^ test, for categorical variables. Binary non-conditional multivariable logistic regression analysis with backward stepwise selection of variables was applied to assess the independent association of presence of DKD, DR, and CVD with age at diabetes diagnosis; covariates were sex (model 1), plus age (model 2), plus other CVD risk factors, i.e., smoking status, PA level, HbA_1c_, BMI, triglycerides, total and HDL cholesterol, systolic and diastolic BP, and anti-hyperglycemic, lipid-lowering, and anti-hypertensive therapy (model 3), plus other complications (model 4). As interdependence of age, age at diabetes diagnosis, and diabetes duration precluded considering all three variables simultaneously, age at diagnosis was included as quartiles instead as a continuous variable in model 5.

Crude mortality rates were described as events per 1000 patient-years from start of follow-up to censoring, with 95% exact Poisson confidence intervals (CIs) and adjusted for age and sex by a Poisson regression model. Kaplan–Meier survival probabilities for all-cause mortality were estimated according to the quartiles of age at diabetes diagnosis and differences were analyzed using the log-rank statistic. The hazard ratios (HRs) and their 95% CIs were estimated by Cox proportional hazards regression with backward selection of variables. These analyses were sequentially adjusted for age and sex (model 1), plus diabetes duration (model 2), or presence of other complications (DKD phenotypes, DR grade, and any CVD), and any severe comorbidity (model 3), or other CVD risk factors (model 4).

All *p* values were two-sided, and a *p* < 0.05 was considered statistically significant. Statistical analyses were performed using SPSS version 13.0 (SPSS Inc., Chicago, IL, USA).

## Results

The baseline clinical features of participants stratified by age at type 2 diabetes diagnosis are shown in Table 1. Individuals falling in quartile I (earliest-onset) were diagnosed at 20.5–45.9 years, thus substantially meeting the criterion for disease onset in early adulthood [10]. Moreover, on average, they were 5.5, 8.2, and 14.7 years younger and had a history of diabetes 4.7, 9.2, and 13.3 years longer, compared with participants falling in quartiles II, III, and IV, respectively. Values of HbA_1c_, BMI, waist circumference, triglycerides, albuminuria, and eGFR as well as proportion of participants of male sex, on insulin treatment, and with a family history of diabetes, current smoking habits, and a moderately/highly active lifestyle decreased from earliest-onset to latest-onset quartile. The opposite was observed for total, HDL, non-HDL, and LDL cholesterol, systolic BP and pulse pressure, and prevalence of hypertension, anti-hypertensive, anti-platelet, and anti-coagulant treatment, any comorbidity, chronic obstructive pulmonary disease, and cancer. The prevalence of albuminuric DKD with preserved eGFR and any, non-advanced and advanced DR decreased, whereas that of both nonalbuminuric and albuminuric DKD with reduced eGFR increased from earliest-onset to latest-onset quartile. Prevalence of any CVD, myocardial infarction, and any coronary event did not differ among quartiles, whereas prevalence of stroke increased and that of the other CVD events decreased from earliest-onset to latest-onset quartile.

**Table 1:** Baseline clinical features of study participants by quartiles of age at type 2 diabetes diagnosis

Variables	I	II	III	IV	*P*
N (%)	3915	3914	3913	3914	
Age at type 2 diabetes diagnosis, years	39.6 ± 4.7	49.8 ± 2.2	57.2 ± 2.2	67.8 ± 5.3	
(range)	(20.5–45.9)	(45.9–53.5)	(53.5–61.2)	(61.2–93.5)	
Age, years	59.5 ± 11.3	65.0 ± 9.5	67.7 ± 7.5	74.2 ± 6.3	< 0.0001
Sex, n (%)					< 0.0001
Females	1524 (38.9)	1661 (42.4)	1717 (43.9)	1852 (47.3)	
Males	2391 (61.1)	2253 (57.6)	2196 (56.1)	2062 (52.7)	
Family history of diabetes, n (%)	1996 (51.0)	1838 (47.0)	1852 (43.5)	1704 (47.3)	< 0.0001
Smoking, n (%)					< 0.0001
Never	2169 (55.4)	2162 (55.2)	2195 (56.1)	2323 (59.4)	
Former	1019 (26.0)	1102 (28.2)	1119 (28.6)	1167 (29.8)	
Current	727 (18.6)	650 (16.6)	599 (15.3)	424 (10.8)	
PA level, n (%)					< 0.0001
inactive or moderately inactive	2449 (62.6)	2604 (66.5)	2439 (62.3)	2452 (62.6)	
moderately active	1369 (35.0)	1255 (32.1)	1417 (36.2)	1435 (36.7)	
highly active	97 (2.5)	55 (1.4)	57 (1.5)	27 (0.7)	
Diabetes duration, years	19.8 ± 10.3	15.1 ± 9.6	10.6 ± 7.5	6.5 ± 5.3	< 0.0001
HbA_1c_, %	7.93 ± 1.57	7.69 ± 1.48	7.41 ± 1.41	7.15 ± 1.43	< 0.0001
BMI, kg·m^−2^	29.2 ± 5.5	29.2 ± 5.3	28.9 ± 5.0	28.6 ± 4.7	< 0.0001
Waist circumference, cm	103.0 ± 11.2	102.9 ± 10.7	102.4 ± 10.1	101.6 ± 9.5	< 0.0001
Obesity, n (%)	1468 (37.5)	1467 (37.5)	1406 (35.9)	1335 (34.1)	0.004
Triglycerides, mmol·l^−1^	1.60 ± 1.15	1.59 ± 0.99	1.57 ± 1.02	1.52 ± 0.79	0.002
Total cholesterol, mmol·l^−1^	4.75 ± 1.01	4.78 ± 0.99	4.79 ± 0.97	4.82 ± 0.99	0.017
HDL cholesterol, mmol·l^−1^	1.27 ± 0.36	1.28 ± 0.35	1.30 ± 0.35	1.31 ± 0.36	< 0.0001
Non-HDL cholesterol, mmol·l^−1^	3.47 ± 0.98	3.50 ± 0.95	3.49 ± 0.93	3.50 ± 0.95	0.436
LDL cholesterol, mmol·l^−1^	2.76 ± 0.85	2.79 ± 0.84	2.79 ± 0.83	2.81 ± 0.85	0.078
Dyslipidaemia, n (%)	3147 (80.4)	3222 (82.3)	3291 (84.1)	3196 (81.7)	< 0.0001
Systolic BP, mmHg	136.6 ± 18.5	138.1 ± 18.0	137.9 ± 17.2	139.7 ± 18.2	< 0.0001
Diastolic BP, mmHg	79.0 ± 9.5	79.0 ± 9.5	78.6 ± 9.3	78.4 ± 9.4	0.012
Pulse pressure, mmHg	57.6 ± 15.8	59.1 ± 15.5	59.2 ± 15.0	61.3 ± 16.2	< 0.0001
Hypertension, n (%)	3053 (78.0)	3272 (83.6)	3310 (84.6)	3461 (88.4)	< 0.0001
Anti-hyperglycaemic treatment, n (%)					< 0.0001
Lifestyle only	341 (8.7)	410 (10.5)	566 (14.5)	796 (20.3)	
Non-insulin	2092 (53.4)	2466 (63.0)	2527 (64.6)	2534 (64.7)	
Insulin	1482 (37.9)	1038 (26.5)	820 (21.0)	584 (14.9)	
Lipid-lowering treatment, n (%)	1764 (45.1)	1781 (45.5)	1884 (48.1)	1809 (46.2)	0.032
Anti-hypertensive treatment, n (%)	2527 (64.5)	2734 (69.9)	2794 (71.4)	3017 (77.1)	< 0.0001
Anti-platelet treatment, n (%)	1434 (36.6)	1529 (39.1)	1578 (40.3)	1707 (43.6)	< 0.0001
Anti-coagulant treatment, n (%)	124 (3.2)	141 (3.6)	147 (3.8)	257 (6.6)	< 0.0001
Albuminuria, mg·day^−1^	79.8 ± 260.8	79.1 ± 427.9	69.1 ± 308.3	61.3 ± 235.9	0.028
Serum creatinine, μmol·l^−1^	88.6 ± 36.9	89.1 ± 33.4	89.1 ± 35.6	91.0 ± 32.6	0.014
eGFR, ml·min^−1^·1.73 m^−2^	86.8 ± 22.2	81.6 ± 20.7	79.7 ± 19.2	73.1 ± 19.2	< 0.0001
Any DKD, n (%)	1337 (34.2)	1368 (35.0)	1370 (40.8)	1597 (35.0)	< 0.0001
DKD phenotype, n (%)					< 0.0001
No DKD	2578 (65.8)	2546 (65.0)	2543 (65.0)	2317 (59.2)	
Albuminuric DKD with preserved eGFR	847 (21.6)	748 (19.1)	739 (18.9)	632 (16.1)	
Nonalbuminuric DKD	214 (5.5)	316 (8.1)	362 (9.3)	584 (14.9)	
Albuminuric DKD with reduced eGFR	276 (7.0)	304 (7.8)	269 (6.9)	381 (9.7)	
Any DR, n (%)	1315 (33.6)	992 (25.3)	681 (12.2)	479 (17.4)	< 0.0001
DR grade, n (%)					< 0.0001
No	2600 (66.4)	2922 (74.7)	3232 (82.6)	3435 (87.8)	
Non-advanced	696 (17.8)	581 (14.8)	401 (10.2)	269 (6.9)	
Advanced	619 (15.8)	411 (10.5)	280 (7.2)	210 (5.4)	
CVD, n (%)					
Any	944 (24.1)	893 (22.8)	880 (22.5)	903 (23.1)	0.354
Myocardial infarction	419 (10.7)	402 (10.3)	460 (11.8)	461 (11.8)	0.077
Coronary revascularization	439 (11.2)	393 (10.0)	393 (10.0)	354 (9.0)	0.017
Any coronary event	615 (15.7)	583 (14.9)	606 (15.5)	592 (15.1)	0.754
Stroke	111 (2.8)	122 (3.1)	110 (2.8)	170 (4.3)	< 0.0001
Carotid revascularization	267 (6.8)	248 (6.3)	191 (4.9)	150 (3.8)	< 0.0001
Any cerebrovascular event	361 (9.2)	344 (8.8)	287 (7.3)	300 (7.7)	0.006
Ulcer/gangrene/amputation	183 (4.7)	150 (3.8)	118 (3.0)	105 (2.7)	< 0.0001
Lower limb revascularization	136 (3.5)	125 (3.2)	106 (2.7)	83 (2.1)	0.002
Any peripheral event	288 (7.4)	239 (6.1)	193 (4.9)	163 (4.2)	< 0.0001
Comorbidities n (%)					
Any	564 (14.4)	646 (16.5)	688 (17.6)	889 (22.7)	< 0.0001
COPD	100 (2.6)	134 (3.4)	152 (3.9)	288 (7.4)	< 0.0001
Chronic liver disease	356 (9.1)	363 (9.3)	313 (8.0)	329 (8.4)	0.155
Cancer	146 (3.7)	226 (5.8)	275 (7.0)	384 (9.8)	< 0.0001

PA = physical activity; HbA_1c_ = hemoglobin A_1c_; BMI = body mass index; BP = blood pressure; eGFR = estimated glomerular filtration rate; DKD = diabetic kidney disease; DR = diabetic retinopathy; CVD = cardiovascular disease; COPD = chronic obstructive pulmonary disease

In the unadjusted and sex-adjusted models, there was a significant association of DKD (positive) and DR (negative), but not CVD, with age at diabetes diagnosis; however, all complications were inversely associated with age at diabetes diagnosis upon further sequential adjustments for age, CVD risk factors, and other complications (Table 2). No association was observed between each complication and quartiles of age at diabetes diagnosis when adjusted for sex, age, and diabetes duration (not shown).

**Table 2:** Association of complications with age at type 2 diabetes diagnosis

Complications	Unadjusted		Model 1		Model 2		Model 3		Model 4	
	OR (95% CI)	*P*	OR (95% CI)	*P*	OR (95% CI)	*P*	OR (95% CI)	*P*	OR (95% CI)	***P***
DKD	1.012 (1.009–1.015)	< 0.0001	1.013 (1.010–1.016)	< 0.0001	0.980 (0.976–0.984)	< 0.0001	0.989 (0.985–0.993)	< 0.0001	0.995 (0.991–0.999)	0.022
DR	0.956 (0.952–0.959)	< 0.0001	0.956 (0.952–0.959)	< 0.0001	0.925 (0.921–0.929)	< 0.0001	0.941 (0.936–0.945)	< 0.0001	0.942 (0.938–0.947)	< 0.0001
CVD	–	–	–	–	0.971 (0.967–0.975)	< 0.0001	0.981 (0.976–0.986)	< 0.0001	0.985 (0.980–0.990)	< 0.0001

Binary non-conditional multivariable logistic regression analysis with backward stepwise selection of variables, unadjusted or adjusted for sex (Model 1), plus age (Model 2), plus other CVD risk factors, i.e., smoking status, PA level, HbA_1c_, BMI, triglycerides, total and HDL cholesterol, systolic and diastolic BP, and anti-hyperglycemic, lipid-lowering, and anti-hypertensive therapy (Model 3), plus other complications (Model 4). DKD = diabetic kidney disease; DR = diabetic retinopathy; CVD = cardiovascular disease; OR = odds ratio; CI = confidence interval

As previously reported, valid information on vital status was retrieved for 15,656 participants (99.3% of the cohort). Of these individuals, 12,054 (76.99%) were alive, whereas 3602 (23.01%) had deceased (follow-up duration: 7.42 ± 2.05 years, death rate: 31.02 per 1000 person-years) [22, 26].

**Fig. 1:**
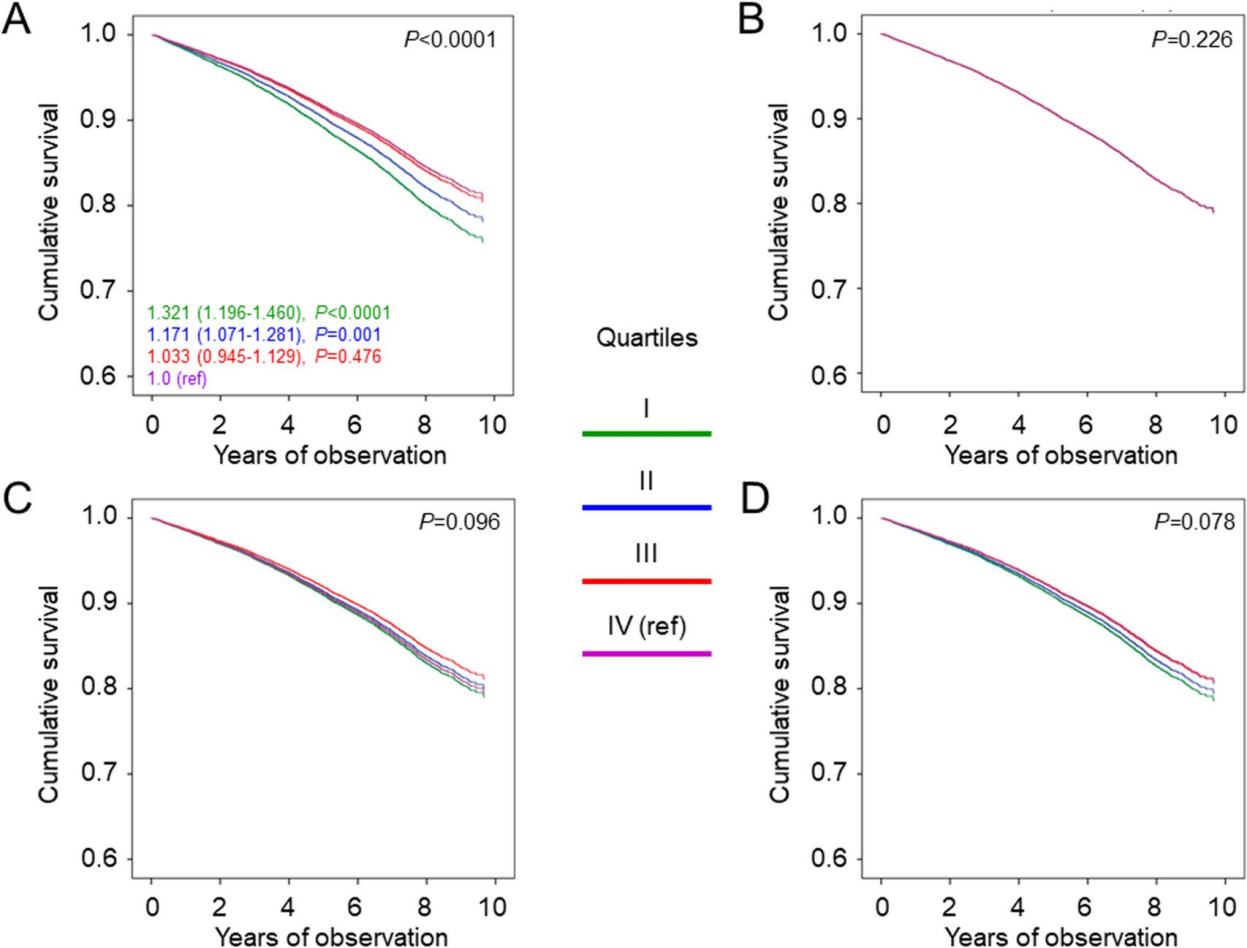
Cox proportional hazards regression by quartiles of age at type 2 diabetes diagnosis, adjusted for age and sex (**A**), plus diabetes duration (**B**), other CVD risk factors (**C**), or complications/comorbidities (**D**). HRs (95% CI) for mortality are shown for each group; quartile I includes patients with earliest-onset diabetes, whereas quartile IV includes patients with latest-onset diabetes. CVD = cardiovascular disease; HR = hazard ratio; CI = confidence interval

Percentages of death, Kaplan–Meier estimates, unadjusted HRs and mortality rates (Supplementary Figure S1A–B and Table 3) increased from earliest-onset to latest-onset quartile, as expected because of the increasing age. In fact, when adjusted for age and sex, participants falling in the earliest-onset quartile showed the highest mortality risk in terms of both HRs (Fig. 1A) and death rates (Table 3). However, differences among quartiles disappeared after further adjustment for diabetes duration, complications/comorbidities, or other CVD risk factors (Fig. 1B–D).

**Table 3:** Mortality rates in study participants by quartiles of age at type 2 diabetes diagnosis

Quartile	N	Events	Percent events	Events per 1,000 patient-years (95% CI), unadjusted	*P*	Events per 1,000 patient-years (95% CI), age- & sex-adjusted	*P*
I	3,915	663	16.9	22.03 (20.41–23.77)	Ref	14.98 (13.07–17.18)	Ref
II	3,914	826	21.1	28.22 (26.36–30.21)	< 0.0001	13.28 (11.64–15.15)	0.023
III	3,913	838	21.4	28.57 (26.70–30.57)	< 0.0001	11.74 (10.29–13.40)	< 0.0001
IV	3,914	1,275	32.6	46.54 (44.06–49.17)	< 0.0001	11.40 (9.99–13.00)	< 0.0001

CI = confidence interval

## Discussion

This analysis of the RIACE cohort showed that type 2 diabetes diagnosed in early adulthood was associated with higher age- and sex-adjusted risk of all-cause death than that occurring later in life. However, no difference was observed when further adjusting for diabetes duration or complications/comorbidities. Of note, despite different prevalence trends across quartiles of age at diabetes diagnosis, all complications were inversely associated with age at disease onset after adjustment for age and sex, but not after further adjustment for diabetes duration.

Our study provides compelling evidence that, in a Caucasian population, the higher mortality risk associated with type 2 diabetes onset in early adulthood is attributable, at least predominantly, to the longer exposure to chronic hyperglycemia (and the other CVD risk factors clustering with it), which results in a higher burden from potentially life-threatening complications such as CVD and DKD. These results add to the existing literature by supporting previous findings obtained in Chinese people with type 2 diabetes. In fact, a prospective study from the Hong Kong Diabetes Registry showed that patients with early versus late disease onset had a higher CVD and DKD risk when adjusting for age and sex, but not when further adjusting for diabetes duration. Moreover, those with early-onset diabetes had a higher and lower cumulative incidence of all-cause mortality, CVD, and DKD at any attained age and disease duration, respectively [5]. Likewise, a cross-sectional survey using data from the China National HbA_1c_ Surveillance System showed that the higher risk of non-fatal CVD in patients with early- versus late-onset type 2 diabetes was greatly attenuated when adjusting for diabetes duration [11]. Another cross-sectional survey in Chinese people participating in the REACTION study showed an increasing risk of CVD events with decreasing category of age at onset, but the effects of diabetes duration was not controlled for [12]. In contrast, a meta-analysis showed that each 1-year increase in age at diabetes diagnosis was associated with a 4% and 6% decreased risk of all-cause mortality and macrovascular and microvascular disease, when adjusting for current age or diabetes duration, respectively; however, these two variables could not be considered simultaneously because of the interdependence with age at diabetes diagnosis [8]. Moreover, a prospective study from the Swedish National Diabetes Registry showed that excess risk for total, CVD, and non-CVD mortality in individuals with type 2 diabetes versus matched non-diabetic controls, adjusted for sex and yearly time-updated duration, with age as the underlying time scale, was highest in patients diagnosed at ≤ 40 years and decreased with each increasing decade of age at diabetes diagnosis [7]. Similar results were obtained in two prospective studies in newly diagnosed individuals with type 2 diabetes from the Kaiser Permanente Northwest Registry [10] and the Kailuan Study [9]. Only in the latter study [9], results were further adjusted for potential confounding from CVD risk factors, though comparison with non-diabetic controls could still be affected by unmeasured confounders preferentially impacting on morbidity and mortality in older individuals. Finally, a study from Hong Kong showed that the adjusted risk for DKD associated with 5-year increase in diabetes duration was higher in people diagnosed at 20–29 years than in those diagnosed at ≥ 70 years, suggesting that early-onset diabetes amplifies the effect of disease duration on risk of DKD [27]. Indeed, a National Registry study from Australia showed that, in the first 10–15 years following type 2 diabetes diagnosis, the incidence of end-stage renal disease was highest in those with late-onset, whereas it became higher in those with early-onset only later, because they were more likely to survive to longer diabetes durations [28].

Differences in age- and sex-adjusted mortality risk among quartiles of age at diabetes diagnosis also disappeared when further adjusting for other CVD risk factors, suggesting that an adverse cardiometabolic risk profile in people with early disease onset may have favored the development of complications driving the increased risk of all-cause death. In fact, participants falling in the lowest quartile had worse glycemic control and higher prevalence of insulin treatment, obesity, atherogenic dyslipidemia, and smoking habits, but lower total and LDL cholesterol, systolic BP and prevalence of other treatments than those falling in the highest quartile. This is consistent with previous reports in patients with type 2 diabetes diagnosed in early adulthood, who were shown to have a worse cardiometabolic risk profile, except for BP, and to be treated more intensively with anti-hyperglycemic agents, but less intensively with cardioprotective drugs [5, 29, 30]. These differences in cardiometabolic risk profile between patients with early- and late-onset type 2 diabetes, which were shown to be present since diagnosis and to persist thereafter [29, 30], may reflect differences in the pathophysiology of the disease or in factors affecting diabetes care and self-management. The worse glycemic control and the greater proportion of insulin-treated individuals in this and other studies [5–7, 9–12, 29, 30] suggest a more severe insulin deficiency, consistent with the more rapid decline in β-cell function reported in early- versus late-onset type 2 diabetes [3, 13], though evidence is mainly derived from studies in individuals diagnosed in childhood or adolescence [31]. Moreover, the higher BMI and waist circumference in participants falling in the lowest versus the other quartiles of age at diagnosis is consistent with the central role of obesity in early disease onset [3, 13, 19], together with family history of type 2 diabetes, the effect of which may be mediated through shared genetic risk and environment favoring obesity [19]. Indeed, while the percentage of patients with grade II or III obesity was higher in the I than in the IV quartile of age at diagnosis (14.1% vs. 9.7%), that of patients with normal weight or under-weight was similar (22.6% vs. 22.4%), suggesting two extreme phenotypes of type 2 diabetes developing in early adulthood, i.e., one driven by severe obesity and the other one resembling the previously described subgroup of adult-onset diabetes in which severe insulin deficiency was associated with lower BMI and higher risk of DR [32], the prevalence of which was in fact highest in the RIACE participants falling in the lowest quartile of age at diagnosis. However, a prospective study showed that individuals with type 2 diabetes aged < 40 years were significantly more likely to be not only obese, but also from minority ethnic groups and the most deprived areas than those aged > 40 years [33], pointing to the importance of socioeconomic factors. Other factors contributing to the more severe disease phenotype when occurring in early adulthood include (a) delayed diagnosis due to a lower likelihood of opportunistic screening; (b) lower adherence to lifestyle recommendations and poorer diabetes self-care practices; and (c) less aggressive treatment owing to the underestimation of risk of complications and lack of specific guidelines, with those tailored for later-onset type 2 diabetes recommending drug prescription based on the assessment of 10-year risk, which is mainly driven by current age [3, 5, 7, 19, 34]. Finally, it was shown that the lower the age at diabetes diagnosis, the higher the prevalence of depression, anxiety and psychological distress [35], which may negatively impact on treatment adherence, thus resulting in worse cardiometabolic risk profile and poorer outcomes.

Strength of our study include the large sample size, the completeness of baseline and follow-up data and, particularly, the assessment of a wide range of clinical parameters which allowed accounting for several confounders. However, there are several limitations. First, the lack of information on the causes of death did not allow detecting differences in CVD versus non-CVD mortality. Second, the study included individuals with prevalent rather than new-onset type 2 diabetes and, hence, participants were not monitored from the time of diagnosis to evaluate trajectories of CVD risk factors and complications. Third, as assay of diabetes-specific auto-antibodies or genetic testing were not performed in all the RIACE participants at the time of diagnosis, it is possible that some cases of autoimmune or monogenic diabetes were misdiagnosed as type 2 diabetes. Fourth, the study findings may not be applicable to the general ambulatory population, as only part of the individuals with type 2 diabetes attend Diabetes Clinics in Italy. Finally, the observational design makes causal interpretation impossible.

## Conclusions

This analysis in Caucasian patients with type 2 diabetes from the RIACE cohort showed that the lower the age at diabetes diagnosis, the higher the age- and sex-adjusted risk of all-cause death. However, the higher mortality risk associated with onset of type 2 diabetes in early adulthood appeared to be mainly driven by the longer disease duration and exposure to chronic hyperglycemia and other CVD risk factors, which explained the increased risk of complications and death. Differences in cardiometabolic risk profile according to age at diagnosis might also be responsible for differences in outcomes, though it is difficult to establish whether they reflect an intrinsically more aggressive disease or factors affecting diabetes care and self-management. These data point to the need for public health policies and tailored guidelines for preventing and treating early-onset type 2 diabetes to avoid premature morbidity and mortality from complications.

## Supplementary Information

Below is the link to the electronic supplementary material.

Supplementary Material (DOCX 571 kb)

## Data Availability

The datasets used and/or analysed during the current study are available from the corresponding author on reasonable request.

## Abbreviations

CVD: Cardiovascular disease
RIACE: Renal Insufficiency and Cardiovascular Events
eGFR: Estimated glomerular filtration rate
PA: Physical activity
BP: Blood pressure
BMI: Body mass index
HbA_1c_: Hemoglobin A_1c_
DKD: Diabetic kidney disease
DR: Diabetic retinopathy
CI: Confidence interval
HR: Hazard ratio
