# Supplementary material for: Association between age at diagnosis and all-cause mortality in type 2 diabetes: the Renal Insufficiency and Cardiovascular Events (RIACE) Italian Multicenter Study
Source: Acta Diabetol. 2024 May 7;61(9):1107–16. doi: 10.1007/s00592-024-02294-1 (PMC11379756; doi:10.1007/s00592-024-02294-1)
Supplement: Supplementary file 1 — Supplementary Material (DOCX 571 kb) [file 592_2024_2294_MOESM1_ESM.docx]

**Supplementary file 1: The RIACE Study Group.**

**The RIACE Steering Committee**

Giuseppe Pugliese (Coordinator), Giuseppe Penno (Secretary), Anna Solini, Enzo Bonora, Emanuela Orsi, Roberto Trevisan, Luigi Laviola, Antonio Nicolucci.

**Participating Diabetes Centers**

1. Azienda Ospedaliera Sant'Andrea, Roma (Coordinating Center): Giuseppe Pugliese, Lucilla Bollanti, Elena Alessi, Martina Vitale, Jonida Haxhi, and Lorenza Mattia.
2. Ospedale Le Molinette, Torino: Paolo Cavallo-Perin, Gabriella Gruden, and Bartolomeo Lorenzati.
3. Ospedale San Luigi Gonzaga, Orbassano: Franco Cavalot, Mariella Trovati, Leonardo Di Martino, and Fabio Mazzaglia.
4. Ospedale San Raffaele, Milano: Giampaolo Zerbini, Valentina Martina, Silvia Maestroni, and Valentina Capuano.
5. IRCCS “Cà Granda – Ospedale Maggiore Policlinico”, Milano: Emanuela Orsi, Valeria Grancini, and Veronica Resi.
6. Ospedale San Paolo, Milano: Antonio Pontiroli, Annamaria Veronelli, and Barbara Zecchini.
7. Ospedale San Giuseppe, Milano: Maura Arosio, Laura Montefusco, Antonio Rossi, and Guido Adda.
8. ASST - Ospedale Papa Giovanni XXIII, Bergamo: Roberto Trevisan, Anna Corsi, and Mascia Albizzi.
9. Ospedale Maggiore, Verona: Enzo Bonora, and Giacomo Zoppini.
10. Policlinico Universitario, Padova: Angelo Avogaro, and Monica Vedovato.
11. Ospedale Cisanello, Azienda Ospedaliero-Universitaria Pisana, Pisa: Giuseppe Penno, Laura Pucci, Daniela Lucchesi, Eleonora Russo, and Monia Garofolo.
12. Ospedale Santa Chiara, Azienda Ospedaliero-Universitaria Pisana, Pisa: Anna Solini.
13. Ospedale Le Scotte, Siena: Francesco Dotta, Cecilia Fondelli, and Laura Nigi.
14. Policlinico Umberto I, Roma: Susanna Morano, Tiziana Filardi, Irene Turinese, and Marco Rossetti.
15. Ospedale S. Maria Goretti, Latina: Raffaella Buzzetti and Chiara Foffi.
16. Ospedali Riuniti, Foggia: Mauro Cignarelli, Olga Lamacchia, Sabina Pinnelli, and Lucia Monaco.
17. Policlinico Universitario, Bari: Francesco Giorgino, Luigi Laviola, and Annalisa Natalicchio.
18. Policlinico Mater Domini, Catanzaro: Giorgio Sesti and Francesco Andreozzi.
19. Policlinico Monserrato, Cagliari: Marco Giorgio Baroni, Giuseppina Frau, and Alessandra Boi.


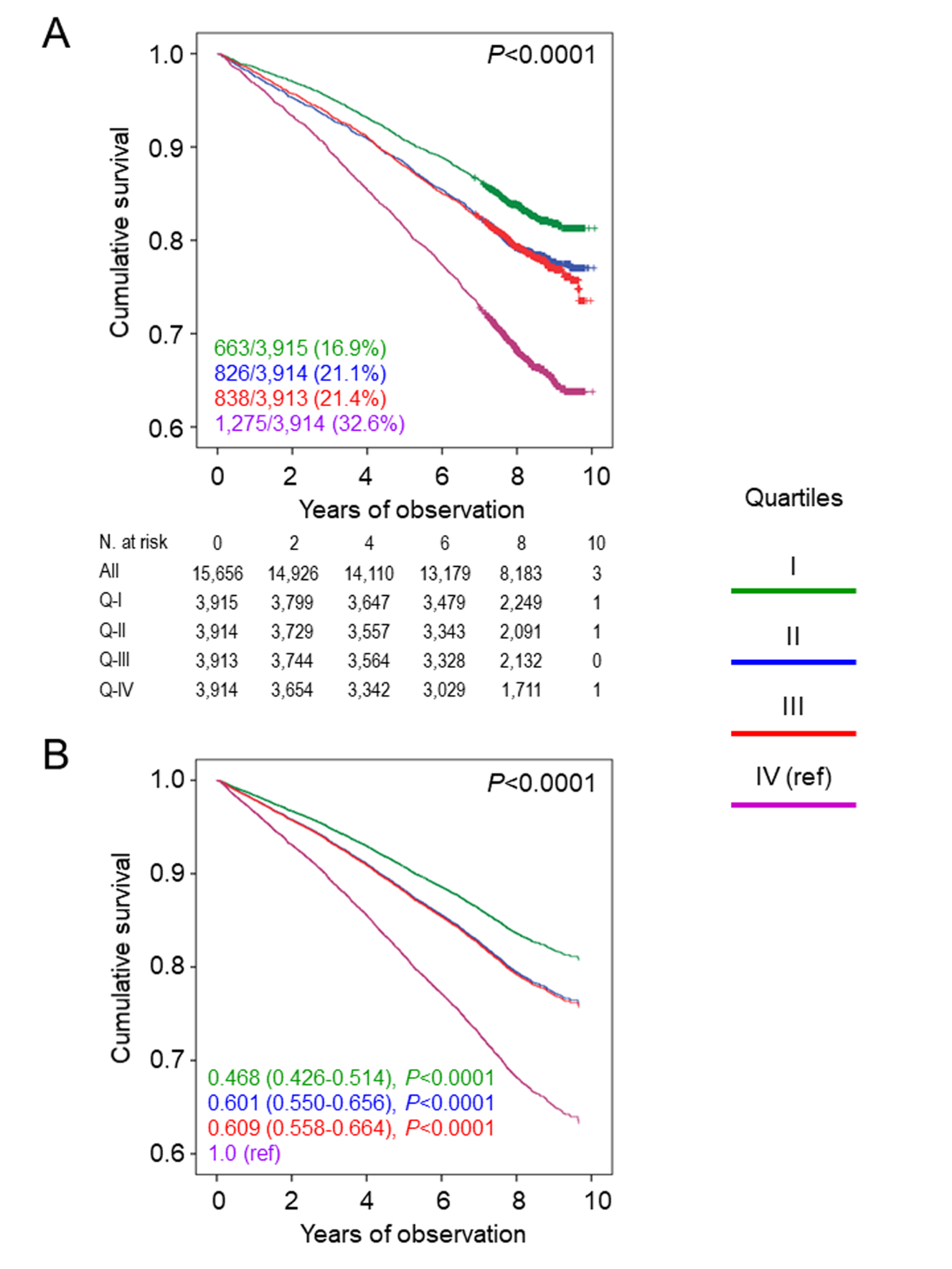


**Supplementary Figure 1.** Kaplan Meier analysis (A) and unadjusted Cox proportional hazards regression (B) by quartiles of age at type 2 diabetes diagnosis. Numbers (percentages) of deaths and HRs (95% CI) for mortality are shown for each group; quartile I includes patients with earliest-onset diabetes, whereas quartile IV includes patients with latest-onset diabetes. HR = hazard ratio; CI = confidence interval.
